# Menstrual Cycle Modulation of Verbal Performance and Hemispheric Asymmetry

**DOI:** 10.3390/brainsci15111141

**Published:** 2025-10-24

**Authors:** Ivana Hromatko, Meri Tadinac

**Affiliations:** Department of Psychology, Faculty of Humanities and Social Sciences, University of Zagreb, Luciceva 3, 10000 Zagreb, Croatia; mtadinac@ffzg.hr

**Keywords:** functional asymmetries, sex hormones, activational effects, verbal processing

## Abstract

**Background/Objectives**: It has been postulated that sex differences in certain types of verbal abilities arise from sex-dimorphic patterns of hemispheric activation, and that these patterns might be modulated by circulating levels of sex hormones. The aim of this study was to explore the activational effects of sex hormones (throughout the menstrual cycle) on both verbal performance and functional hemispheric asymmetries (qEEG laterality) in three types of verbal tasks: sex-differentiated (verbal fluency and semantic decision) vs. sex-neutral (verbal reasoning) tasks. **Methods**: A group (*n* = 32) of healthy young women was tested twice, once during the mid-luteal (high levels of circulating sex hormones) and once during the early follicular (low levels of sex hormones) phases of the menstrual cycle. A comparable group of healthy young men (*n* = 32) was tested once. EEG was continuously recorded. The differences in alpha power on homologous sites of the left and right hemispheres were then calculated. **Results**: We found a clear congruence between performance on a task and laterality score: for sex-differentiated tasks, the activational effects of sex hormones were observed in both performance and laterality scores, while there were neither performance nor laterality scores shifts throughout the menstrual cycle for the sex-neutral task. Interestingly, measures of functional asymmetry were higher in the luteal compared to the menstrual phase. **Conclusions**: These findings suggest that sex hormones modulate verbal performance through their influence on hemispheric asymmetry.

## 1. Introduction

Sex differences in verbal and spatial abilities are among the most extensively studied interindividual differences in psychology, and they have attracted scholarly attention for decades [1,2,3,4,5,6,7]. These findings are often discussed in relation to functional hemispheric asymmetries, as sex differences in brain lateralization are thought to contribute to performance discrepancies in sex-differentiated tasks. Although such sex differences in hemispheric asymmetry are usually small, they are statistically robust and consistently reported in relation to verbal and spatial task performance [8,9,10]. Sex hormones influence neural structures involved in cognition, both prenatally (organizational effects) and across the lifespan (activational effects), where circulating hormone levels modulate neural circuits and correlate with cognitive performance [11,12,13,14,15]. Elevated estrogen and progesterone levels generally enhance left-hemisphere functions (e.g., verbal abilities) but can impair right-hemisphere functions (e.g., spatial tasks). If women’s advantage in verbal tasks is linked to greater interhemispheric communication [16], it follows that hormonal fluctuations may alter laterality by modulating interhemispheric transfer. This framework explains the recurring finding that women often perform better on “male-typical” tasks during low-hormone phases of the menstrual cycle.

Hampson [11,12] compared the performance of participants in the menstrual and luteal phases of the menstrual cycle across several verbal tasks: a verbal fluency test (generating as many words as possible beginning with a given letter in limited time), an expressive fluency test (creating as many sentences as possible of a given length, with all words starting with the same letter), oral articulation tasks (counting aloud, reading aloud a list of color names, and naming sequences of colored dots), and rapid repetition of syllables (e.g., ba, da, and ga). In all tasks, performance was better in the mid-luteal and late follicular phases than in the early follicular (menstrual) phase.

In postmenopausal women receiving estrogen therapy, synthetic estrogens also showed a positive effect on cognitive functioning. Improvements were observed across all domains, including spatial ability, suggesting that estrogen in later life may have a protective role in maintaining intellectual functioning and memory [13,14].

EEG coherence analyses during verbal tasks suggest that the classic view of strongly lateralized language functions should be revised. Weiss and Müller [17] argue that the traditional Wernicke–Geschwind model must be expanded: language functions are both localized and distributed, relying on dynamic information exchange among multiple regions that are important but not exclusively language-specific. Activation of language areas alone does not ensure intact function, pointing to the need for “hybrid” neurophysiological models.

Khateb et al. [18], using a divided visual field paradigm with ERP recordings, found that even when a semantic task was presented to the right hemisphere, information transfer to the left hemisphere occurred within just 150 ms. Their findings revealed a neural network for verbal material processing that included the superior occipital gyrus, middle and inferior temporal gyri, the left inferior frontal gyrus, and also regions of the right hemisphere. Other studies confirmed the link between asymmetries and performance. For example, Davidson et al. [19] showed that EEG parietal and central, but not frontal asymmetries, positively correlate with cognitive performance. Resting-state EEG measures similarly highlight the functional importance of asymmetry, as spontaneous cortical activity influences perception and cognition [20,21]. Prefrontal asymmetries predict affective evaluation efficiency [22,23,24], while variations in cognitive performance relate only to posterior cortical asymmetries during task activation [22,25]. Nettle [26] further demonstrated that the degree, not the direction, of lateralization matters—explaining findings that right-handers appear more cognitively efficient simply because of stronger lateralization. EEG asymmetries thus reflect tonic hemispheric activation patterns underlying cognitive processes.

Functional MRI studies also reveal sex-specific activation patterns. Gizewski et al. [27] found that during mental rotation, men showed increased activation in the right medial frontal and precentral gyri and bilateral inferior parietal cortex, whereas women showed stronger activation in the right inferior and medial temporal gyri, right superior frontal gyrus, and left fusiform gyrus. For verbal fluency tasks, sex differences were minimal, with women showing greater activation in the left medial temporal and precentral gyri. Thus, larger sex-related differences appeared for mental rotation than for word generation.

Across studies on hormonal influences, elevated estrogen and progesterone levels generally enhance functions associated with the left hemisphere while impairing those linked to the right. If men’s superior spatial performance stems from stronger right-hemisphere specialization, and women’s superior verbal performance stems from greater interhemispheric coordination [16], then hormonal fluctuations may alter both the degree of lateralization and the extent of interhemispheric communication. Indeed, many findings suggest that higher circulating levels of sex hormones reduce asymmetry, while lower levels increase it, consistent with women performing better on sex-differentiated tasks, favoring men during low-hormone phases.

Hausmann and Güntürkün [28], using a divided visual field paradigm, demonstrated that lateralization varies with the menstrual cycle. Left-hemisphere dominance for verbal processing was weaker mid-cycle than during menstruation (when levels of circulating estrogen and progesterone are lower), and lateralization was stronger. Similarly, smaller asymmetries were found for spatial tasks in the luteal phase of the menstrual cycle. In postmenopausal women, lateralization levels resembled those of men and younger menstruating women. The authors argued that progesterone was key: higher levels appeared to stimulate the non-dominant hemisphere for spatial tasks, reducing asymmetry, a process they termed hemispheric decoupling. In a follow-up study, Hausmann et al. [29] tracked women with natural cycles over six weeks, measuring hormone levels and lexical decision performance. Only progesterone correlated with asymmetry, whereas estradiol influenced both hemispheres equally. Their “decoupling hypothesis” posits that progesterone reduces glutamate effects on non-NMDA receptors, diminishing interhemispheric transfer and thus functional asymmetries. However, later studies did not confirm this mechanism [30].

Stimuli in divided visual field paradigms may be too simple to generalize to complex tasks, yet surprisingly few studies have examined whether hormonal effects on cognition are observable with neuroimaging. Fernández et al. [31] used fMRI and serum hormone assays while participants performed a perceptual speed task (symbol comparison) and a semantic task (synonym judgment). Behavioral performance did not differ between menstrual and luteal phases, but activation patterns did: the semantic task elicited dominant left-hemisphere activation (frontal and temporo-parietal areas), while the perceptual speed task activated bilateral posterior regions. Progesterone correlated with activation in the superior temporal gyrus, and both progesterone and estradiol correlated with medial superior frontal activation bilaterally. Further evidence comes from Weis et al. [32], who used fMRI to show that during a word-matching task, inhibitory effects of left-hemisphere language regions on right-hemisphere homologues were strongest during menstruation, resulting in greater lateralization. In the follicular phase, inhibition was reduced, decreasing asymmetry, demonstrating estradiol’s neuromodulatory role in functional brain organization.

In general, desynchronization in the lower alpha band (8–10 Hz) reflects changes in attention [33,34] and working memory [35]. EEG coherence studies confirm greater regional activation and interhemispheric coordination in women during verbal tasks compared to rest [36]. Given that interindividual EEG differences exceed intraindividual ones [22] and that alpha power and coherence measures remain stable across cycle phases [37], repeated-measures designs with the same participants are justified.

Taken together, these findings suggest that the activational effects of sex hormones influence hemispheric asymmetry and interhemispheric communication, particularly in tasks with known sex-differentiation. Yet, relatively few studies have investigated these mechanisms using neuroimaging or EEG. This motivates further research into whether EEG activation patterns are indeed sex- and cycle-specific across both sex-differentiated tasks (e.g., verbal fluency, where women excel) and sex-neutral tasks (e.g., verbal reasoning). Hence, the aim of this study was to investigate the activational effects of sex hormones across the menstrual cycle on verbal cognitive performance and associated functional hemispheric asymmetries, as measured using quantitative EEG laterality indices. Specifically, we examined performance in three types of verbal tasks that differ in their sensitivity to sex effects: two sex-differentiated tasks—verbal fluency and semantic decision, which typically show female advantages—and one sex-neutral task, verbal reasoning, for which neither sex differences nor menstrual cycle-related hormonal effects were expected. By comparing behavioral outcomes and hemispheric activation patterns across the early follicular (low estrogen and progesterone) and mid-luteal (high estrogen and progesterone) phases of the menstrual cycle, we aimed to determine whether cyclical hormonal fluctuations are associated with changes in task performance and lateralized brain activity, and whether these effects are specific to tasks with known sex differentiation. More specifically, we formulated two sets of hypotheses:

1. Behavioral performance: We expected task performance to vary as a function of menstrual cycle phase only for the sex-differentiated tasks (verbal fluency and semantic decision), but not for the sex-neutral task (verbal reasoning). Specifically, we hypothesized that women would perform better during the mid-luteal phase (characterized by high estrogen and progesterone levels) compared to the early follicular phase (low hormone levels).
2. Neural asymmetries: We further hypothesized that changes in hemispheric activation patterns, as reflected in EEG asymmetry indices, would occur across menstrual cycle phases only for the sex-differentiated tasks (verbal fluency and semantic decision), but not for the sex-neutral task (verbal reasoning).

## 2. Materials and Methods

### 2.1. Participants and Procedure

The research was conducted in the facilities of the Department of Psychology, Faculty of Humanities and Social Sciences in Zagreb. The study adhered to the Declaration of Helsinki and received approval from the Ethics Committee of the Department of Psychology, Faculty of Humanities and Social Sciences, University of Zagreb (approval code: EPOP_2021_22_13).

Eligibility criteria included: regular menstrual cycles (26–32 days), right-handedness, absence of medical conditions that could affect EEG recordings (e.g., epilepsy, concussions, and neurological disorders), and no use of oral contraceptives within six months prior to participation. Women not meeting these criteria were excluded. Health status and cycle regularity were verified through a questionnaire at enrollment.

Initially, 39 healthy women (age range 21–33 years; *M* = 23.8, *SD* = 2.6) with natural menstrual cycles were recruited. Five participants were excluded from the analyses because follow-up information about the onset of their next menstrual cycle revealed that at least one measurement had been taken at an incorrect cycle phase (details on cycle phase determination are provided below), and data from two participants were excluded due to excessive noise in their EEG recordings, which made analyses unreliable. To ensure equal sample sizes for men and women, we recruited 32 male participants (age range 20–31 years; *M* = 22.6, *SD* = 2.3). Men were tested once: to minimize potential confounds related to diurnal testosterone fluctuations, all male participants were tested within the same time window (10:00–12:00 a.m.). Women were tested twice—once in the early follicular phase (characterized by low estradiol and progesterone) and once in the mid-luteal phase (characterized by high estradiol and progesterone)—with testing order counterbalanced.

Testing dates were determined individually using the backward-counting method, which estimates ovulation by subtracting 14 days from the expected onset of the next menses. Compared to forward-counting, this method provides greater validity when relying on self-reports (0.77 vs. 0.40–0.55) [38,39]. For each participant, the early follicular session was scheduled on cycle days 3–5 (excluding days 1–2 to avoid potential effects of menstrual discomfort), and the mid-luteal session was scheduled 5–8 days prior to the anticipated onset of the next cycle. Participants were later contacted to confirm the actual start date of their next menstruation.

EEG was recorded using a Nihon Kohden electroencephalograph (model Neurofax-9200; Tokyo, Japan) with electrodes positioned according to the international 10–20 system, referencing Cz and linked earlobes. Impedances were kept below 5 kΩ. Because central and linked-earlobe referencing has been criticized as unsuitable for calculating asymmetry indices [37], all signals were re-referenced offline using the grand average reference algorithm. This procedure was chosen because averaging across electrodes approximates a neutral reference and minimizes bias from any single site. To confirm that our findings were not reference-dependent, we re-ran all asymmetry analyses using Cz as the reference; the results remained essentially unchanged. Data were band-pass filtered between 0.3–30 Hz.

Tasks were presented on a laptop with E-Prime version 2.0 software installed [40], which generated verbal reasoning tasks and recorded participants’ responses. Event markers were entered into the EEG log each time a task was presented and each time the participant responded. Artifacts caused by noise, eye movements, or muscle activity were removed, and artifact-free epochs of at least 3 s (512 data points) during stimulus presentation were retained. These segments were subjected to fast Fourier transformation to obtain spectral power.

Subsequent analyses were conducted in SPSS v25. As a measure of functional asymmetry, we calculated lateralization indices for the lower alpha band (8–10 Hz) as ln(R) − ln(L), where R and L represent power at homologous right and left electrode sites (F3/F4, F7/F8, T3/T4, T5/T6, and P3/P4). Indices were subsequently pooled across the frontal, temporal, and parietal regions. Because lower alpha power reflects greater cortical activation (i.e., greater suppression), a negative asymmetry value (lnR < lnL) indicates greater right-hemisphere activation, whereas a positive asymmetry value indicates greater left-hemisphere activation. The rationale for choosing the lower alpha band was that it has previously been shown that this frequency bandwidth is sensitive to changes in attention and working memory load [33,34,35].

### 2.2. Tasks and Questionnaires

#### 2.2.1. Verbal Reasoning Task

Each item consisted of a sequence of words that shared a common feature, and among the four answer choices, only one word shared that same feature with the given sequence (e.g., given the sequence salty, sour, sweet and the response options bitter, sharp, intoxicating, fragrant, the correct answer was bitter, as it was the only term referring to taste, like the items in the sequence). The participants were instructed to respond by pressing keys “1,” “2,” “3,” or “4,” depending on which option they considered correct. A total of six items of this type were presented. They were chosen from a larger pool of previously tested items and showed an internal consistency of 0.78.

#### 2.2.2. Semantic Task

This task followed a similar response format. Here, pairs of words were presented on the screen, and participants had to decide whether the words were synonyms. Responses were made by pressing the “S” key if they judged the pair to be synonymous, or the “D” key if they considered the meanings to differ. A total of 12 pairs were presented. The words were deliberately simple and familiar (e.g., bird–chirp or sorrow–misery), as the goal was to activate verbal processing rather than to assess vocabulary knowledge. Because of that, the main performance measure was the time needed to make the semantic decision (in seconds).

#### 2.2.3. Verbal Fluency

Participants completed a sentence generation task in which all words had to begin with a designated letter. After hearing the target letter, they had up to two minutes to prepare the sentence silently and then produce it aloud. This delay minimized interference with the EEG recording. Two sentences were generated in each session: in the first session, with the letters “A” and “K,” and in the second, with “P” and “L.” The number of words per sentence served as the measure of verbal fluency. All EEG epochs were included in the analyses; however, for comparisons of verbal output across menstrual cycle phases, only sentences beginning with “K” and “P” were analyzed, as independent-sample analyses showed that sentences beginning with “A” contained significantly more words than the other letters.

#### 2.2.4. The Mood (a Control Variable)

To control for potential mood fluctuations that could act as confounding variables, the Adjective Check List was administered [41,42]. This questionnaire comprises 52 adjectives describing emotional states. Participants had to respond according to how they felt that day, on a scale ranging from 0 (“Not at all”) to 4 (“Extremely”). Average scores were computed for eight dimensions: anxiety (6 items), depression (6 items), friendliness (5 items), joy (5 items), fatigue (8 items), hostility (8 items), focus (8 items), and energy (6 items). The alpha coefficients of internal consistency for all subscales ranged from 0.84 to 0.95.

## 3. Results

### 3.1. Cognitive Performance

Since previous research has shown that performance on cognitive tests, as well as certain neural asymmetries (e.g., frontal alpha asymmetry), correlate with mood, we first examined whether our participants showed any mood changes across the menstrual cycle. As presented in Table 1, no significant differences were found in any of the eight mood dimensions between the early follicular and mid-luteal phases, with the possible distinction of fatigue, which was slightly higher in the early follicular compared to the mid-luteal phase. For this reason—to control for possible effects of fatigue—all of the analyses involving the performance on the tasks were conducted with fatigue as a covariate.

**Figure 1:**
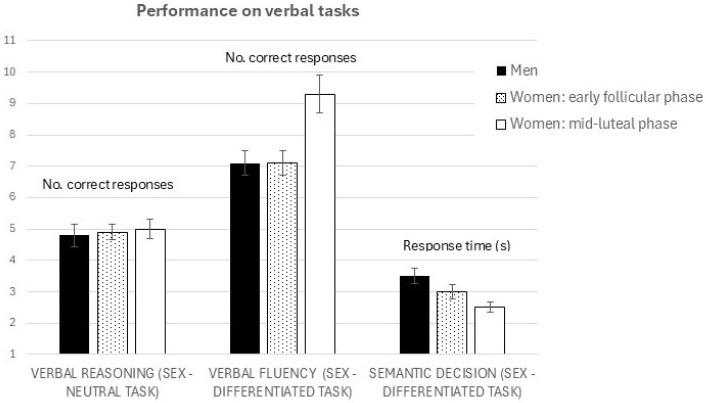
Performance in several verbal tasks, depending on sex and menstrual cycle phase. The scores in the verbal reasoning task pertain to the total number of correctly solved items, the scores in the verbal fluency task pertain to the total number of words in the produced sentence, and the performance in the semantic decision task is expressed average response time (seconds) needed to reach the decision in each item.

Next, repeated-measures ANCOVAs were performed with menstrual cycle phase (early follicular vs. mid-luteal) as the within-subjects source of variance, order of testing (early follicular first vs. mid-luteal first) as the between-subjects source of variance, fatigue scores as covariates, and the behavioral measures as dependent variables. The assumptions of normality and sphericity were met. In cases where Greenhouse–Geisser corrections were applied, the results remained identical to those obtained without the correction. Therefore, only the uncorrected results are reported for clarity. Neither order of testing nor covariates had a significant effect on any of the dependent variables (full tables shown in Supplementary Material, Table S1). Main effects of sex and menstrual cycle followed the expected pattern: Figure 1 shows the scores on verbal performance tasks, depending on sex, and for women, menstrual cycle phase. Regarding verbal reasoning—a sex-neutral task in which neither sex differences nor the activational effects of sex hormones were expected—there was no effect of cycle phase on verbal reasoning accuracy (*F* = 0.87, *p* = 0.357), and no sex differences (*F* = 0.012, *p* = 0.911).

The other two tasks showed the expected sex differences, as well as menstrual cycle-related shifts in performance (activational effects of sex hormones). Women produced significantly longer sentences in mid-luteal (high levels of sex hormones) than in early follicular phase of cycle (*F* = 4.76, *p* = 0.037, ηp^2^ = 0.141) and were on average more verbally fluent than men (comparison made between men and women during their first session only (*F* = 3.95, *p* = 0.05, d = 0.5) Similar pattern was observed for semantic decision, another sex- differentiated task, which showed menstrual-cycle related shifts in time needed to make the semantic decision: women reached (correct) semantic decision faster in mid-luteal phase compared to early follicular (*F* = 5.49, *p* = 0.026, ηp^2^ = 0.164), and were on average faster than men (*F* = 4.84, *p* = 0.031, d = 0.55).

### 3.2. Hemispheric Asymmetries

**Figure 2:**
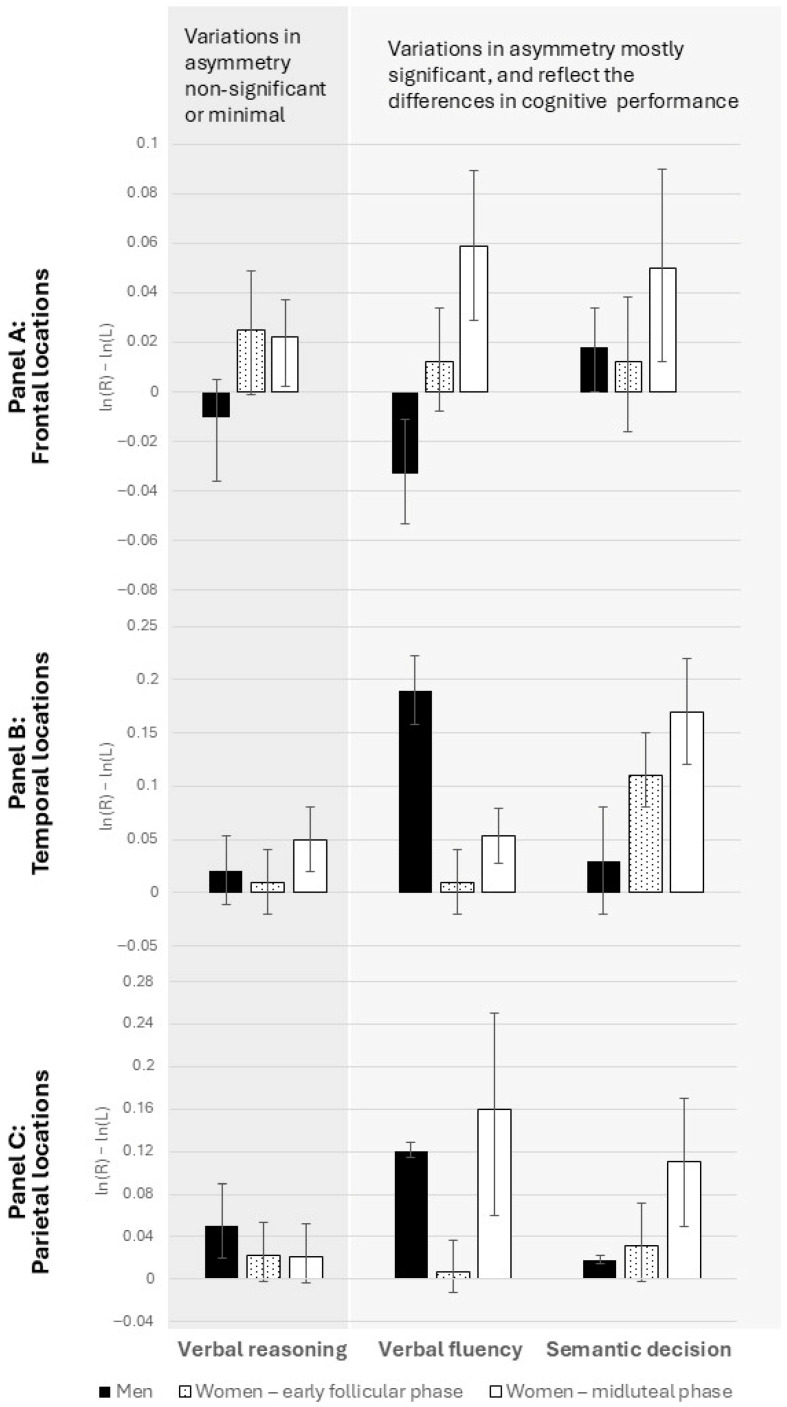
Laterality indices while participants were solving three different verbal tasks: a verbal reasoning task (a sex-neutral task), a verbal fluency task (a sex-differentiated task, favoring women), and a semantic decision task (a sex-differentiated task, favoring women). The scores were derived as differences in alpha power on homologous pairs of electrodes on right—left hemisphere locations and then pooled across regions: (**A**) frontal locations (F3/F4, F7/F8); (**B**) temporal locations (T3/T4, T5/T6); (**C**) parietal locations (P3/P4). Negative asymmetry values indicate greater activation of the right hemisphere compared to the left. Error bars represent 95% CIs.

Repeated-measures ANCOVAs were conducted with menstrual cycle phase (early follicular vs. mid-luteal) as the within-subjects source of variance, baseline EEG activity (without cognitive load) as covariates, and laterality indices as the dependent variables. Table 2, Table 3 and Table 4 show the results of these analyses for verbal reasoning, semantic decision, and verbal fluency, respectively. Figure 2 displays pooled laterality indices across frontal, temporal, and parietal regions during solving the tasks.

For verbal reasoning, results showed a divergence by sex at frontal sites: men exhibited greater right-hemisphere activation, whereas women showed greater left-hemisphere activation (F_front_ = 2.9, *p* < 0.05). However, laterality indices did not vary across menstrual cycle phases (Figure 2, Table 2). At temporal and parietal sites, neither menstrual-cycle-related shifts nor sex differences (*F*_temp_ = 0.5; *F*_par_ = 0.09; *p* > 0.05) were observed. The absence of significant differences aligns with the lack of sex or cycle-related variation in task performance.

During semantic decision-making, both sex differences (*F*_temp_ = 2.6; *F*_par_ = 2.4; *p* < 0.05) and menstrual cycle-related changes (Figure 2, Table 3) emerged at temporal and parietal sites. Frontal sites also showed cycle-related shifts, though sex differences did not reach significance.

Finally, in the verbal fluency task, both sex differences (*F*_front_ = 1.8; *F*_temp_ = 3.9; *F*_par_ = 4.12; *p* < 0.05) and menstrual-cycle-related shifts (Figure 2, Table 4) were observed consistently across all regions. This pattern is consistent with the observed sex differences and menstrual cycle-related shifts in cognitive performance on these tasks.

For a conservative post hoc power estimate for the within-subject comparisons (two time points, *N* = 32) we conducted post hoc power analyses using Cohen’s dz converted from the observed partial η^2^ and a repeated measures correlation of *r* = 0.04. The smallest observed effect (ηp^2^ = 0.14) corresponds to dz = 0.397. Power to detect ηp^2^ ≥ 0.14 at α = 0.05 was sufficient (≈0.98).

## 4. Discussion

The present study examined whether menstrual cycle-related fluctuations in sex hormones are associated with changes in hemispheric asymmetries (qEEG laterality indices) during the performance of verbal tasks differing in their degree of sex bias. Overall, the findings suggest that functional hemispheric asymmetries are not uniformly sensitive to cycle phase but instead show task- and region-specific patterns, which in turn mirror the presence or absence of cycle-related changes in behavioral performance.

For the verbal reasoning task, which was expected to be sex-neutral, the absence of significant menstrual-cycle-related differences in cognitive performance was paralleled by stable laterality indices across frontal, temporal, and parietal regions. The only robust effect was a general sex difference at frontal sites, with men exhibiting relatively greater right-hemisphere activation and women greater left-hemisphere activation. Crucially, these asymmetries did not vary with cycle phase, reinforcing the behavioral finding that verbal reasoning is largely unaffected by the activational effects of sex hormones. It should also be noted that the asymmetries observed during verbal reasoning were very small, which is consistent with the idea that this function is not lateralized. Given that general intelligence is not lateralized, these changes in alpha frequency may reflect altered attentional or memory demands, possibly compensatory, which explains why no performance differences were found.

In contrast, for the semantic decision task, where sex differences were anticipated, both behavioral and neural data pointed to sensitivity to cycle phase. Women in the mid-luteal phase were faster in reaching semantic decisions, and this improvement was accompanied by measurable shifts in laterality at temporal and parietal locations. These findings highlight the role of posterior cortical regions in supporting semantic processing and suggest that hormone-driven changes in interhemispheric dynamics may underlie cycle-related improvements in efficiency. Frontal regions also showed menstrual cycle-related shifts, although these were less clearly tied to behavioral advantages, implying that the cycle’s impact on frontal asymmetries may not directly translate into measurable performance differences on this task.

Finally, the verbal fluency task showed the strongest evidence of hormone-related modulation, both behaviorally and neurally. Women produced longer sentences in the mid-luteal phase, and this enhancement was consistently mirrored by increased laterality shifts across frontal, temporal, and parietal locations. These widespread changes suggest that verbal fluency draws on a distributed cortical network and that the activational effects of sex hormones may amplify interhemispheric coordination across multiple regions simultaneously. Unlike in semantic decision, where posterior regions seemed most sensitive, here both anterior and posterior sites contributed, underscoring the broad neural underpinnings of fluency.

Taken together, these findings underscore a tight correspondence between cognitive performance and hemispheric laterality shifts. When performance remained stable (verbal reasoning), neural asymmetries also remained unchanged. When performance varied with menstrual cycle phase (semantic decision and verbal fluency), corresponding changes in laterality emerged, particularly in regions most relevant for each task (posterior sites for semantic processing, broader networks for fluency). This alignment strengthens the interpretation that observed EEG asymmetries are not epiphenomenal but rather reflect underlying functional changes in hemispheric involvement driven by hormonal fluctuations.

We selected these specific verbal tasks because previous research has shown that they are sensitive to both sex differences and the activational effects of sex hormones—particularly verbal fluency, which has consistently demonstrated such effects. In contrast, verbal reasoning has repeatedly been found to be relatively unaffected by either sex or hormonal influences, and was therefore included as a control task. Although the number and type of tasks in this study were limited, we expect that, following this rationale, verbally based tasks showing sex-related differences would mirror fluency, whereas those insensitive to sex or menstrual-cycle phase would resemble reasoning. More generally, this pattern supports the view that the activational effects of sex hormones selectively modulate verbal processes that rely on flexible retrieval and lexical access, while leaving more abstract, rule-based reasoning unaffected.

The results also highlight that not all cortical regions respond equally: frontal sites consistently showed sex differences in activation, while temporal and parietal sites were particularly sensitive to cycle-related shifts. This suggests that posterior regions might be more dynamically modulated by hormonal state. Although the luteal phase has previously been associated with reduced functional hemispheric asymmetry [11,12,28,29], in these tasks, we observed greater asymmetry indices in the luteal than in the menstrual phase (with relatively greater left-hemisphere activation in both). This finding supports the hypothesis that the direction of asymmetry is less relevant—what matters is the presence of an asymmetry, which enhances cognitive performance [26]. However, it should be noted that the functional interpretation of hemispheric asymmetry remains debated. Our study design does not allow us to conclude that increased asymmetry necessarily implies improved performance, as both the direction and magnitude of asymmetry may reflect different, or even compensatory, neural mechanisms.

From a broader perspective, these findings contribute to ongoing debates about the neurocognitive mechanisms through which sex hormones shape cognition. Demonstrating that cycle-related hormonal shifts selectively modulate hemispheric asymmetry—and that these neural changes align with behavioral outcomes—offers additional evidence for a functional link between activational hormonal effects and interhemispheric coordination. This has implications not only for understanding sex differences in verbal abilities but also for clinical contexts, where fluctuations in estrogen and progesterone have been linked to mood and cognitive disturbances. For example, in PMDD, mood symptoms in the luteal phase have been linked with shifts in frontal alpha EEG asymmetry (PMDD vs. controls) that correspond to negative affect and depressive mood induction [43]. By showing that hemispheric laterality is a sensitive neural marker of hormone-driven changes, the present study provides a potential bridge between basic neuroscience and applied domains such as neuropsychiatry and women’s health.

### Limitations of the Study

This study has several limitations that should be acknowledged. As is common in EEG research, particularly with repeated-measures designs, the sample size was relatively modest, which may limit the generalizability and robustness of the findings. A larger sample would help capture interindividual variability, both in hormonal profiles across the menstrual cycle and in the cognitive strategies employed during verbal tasks. Furthermore, the modest sample size inevitably limits the statistical power to detect complex interactions, such as those between sex and cycle phase. Thus, the lack of significant interaction effects should be interpreted cautiously, as subtle effects may have gone undetected. Another limitation concerns the timing of testing: participants were assessed only during the early follicular and mid-luteal phases. Inclusion of the late follicular phase—marked by high estrogen but low progesterone levels—would have allowed for clearer differentiation between estrogenic and progestogenic effects on verbal performance and hemispheric laterality. Additionally, because objective hormonal measures were not collected, phase determination relied on self-reports, which may introduce variability. Furthermore, the relatively low spatial resolution of EEG constrains the ability to identify the precise neural sources of hormonal effects, as we were limited to examining laterality indices across homologous scalp locations. Future studies using neuroimaging techniques with higher spatial resolution could provide more detailed insights into the neural mechanisms underlying these cycle-related shifts. Finally, given the complexity of the experimental setup and the feasibility constraints associated with both the task and EEG recording, we opted for a smaller set of tasks than would be ideal for a comprehensive neuropsychological assessment. A more extensive and diverse range of verbal tasks would likely provide a richer picture of the underlying cognitive processes.

## 5. Conclusions

The present study demonstrates that hemispheric laterality in verbal tasks varies as a function of both sex and menstrual cycle phase, with shifts in EEG asymmetries paralleling changes in cognitive performance. While verbal reasoning showed minimal asymmetries—supporting its sex-neutral, non-lateralized nature—tasks that rely on flexible retrieval and lexical access (verbal fluency and semantic decision) revealed both sex differences and cycle-related modulations, particularly in temporal and parietal regions. These findings suggest that the activational effects of sex hormones influence not only verbal performance but also its underlying neural activation, highlighting the dynamic interaction between biological states and cognitive processes.

## Figures and Tables

**Table 1:** The results of repeated measures ANOVAs, with menstrual cycle phase as a source of variance and the dimensions of mood as dependent as dependent variables.

	Early Follicular Phase		Mid-Luteal Phase				
	*M*	*SD*	*M*	*SD*	*F*	*p*	ηp^2^
Anxiety	4.97	4.23	4.35	4.00	0.06	0.82	0.002
Depression	5.48	4.67	4.15	3.80	0.62	0.44	0.021
Friendliness	14.37	4.58	14.30	4.03	0.02	0.89	0.001
Joy	11.07	4.35	11.03	4.04	0.01	0.97	0.001
Fatigue	11.67	7.49	8.43	5.92	4.61	0.04	0.137
Hostility	5.58	2.77	4.73	3.30	0.37	0.55	0.013
Focus	20.30	5.72	20.83	5.27	0.33	0.57	0.011
Energy	10.17	4.67	11.17	3.66	1.69	0.20	0.055

**Table 2:** The results of repeated-measures ANCOVAs with menstrual cycle phase (early follicular vs. mid-luteal) as the within-subjects source of variance, baseline EEG activity (without cognitive load) during both phases of the menstrual cycle as covariates, and laterality indices as the dependent variables during the verbal reasoning task.

Task: Verbal Reasoning					
Frontal	Source	*df*	*F*	*p*	ηp^2^
	Menstrual cycle phase	1	0.011	0.918	0.001
	Mid-luteal baseline	1	2.016	0.173	0.101
	Early follicular baseline	1	3.555	0.076	0.165
	Error (cycle)	29			
Temporal	Source	*df*	*F*	*p*	ηp^2^
	Menstrual cycle phase	1	2.732	0.117	0.138
	Mid-luteal baseline	1	0.230	0.638	0.013
	Early follicular baseline	1	1.825	0.194	0.097
	Error (cycle)	29			
Parietal	Source	*df*	*F*	*p*	ηp^2^
	Menstrual cycle phase	1	0.002	0.969	0.000
	Mid-luteal baseline	1	0.007	0.935	0.000
	Early follicular baseline	1	0.111	0.742	0.006
	Error (cycle)	29			

**Table 3:** The results of repeated-measures ANCOVAs with menstrual cycle phase (early follicular vs. mid-luteal) as the within-subjects source of variance, baseline EEG activity (without cognitive load) during both phases of the menstrual cycle as covariates, and laterality indices as the dependent variables during the semantic decision task.

Task: Semantic Decision					
Frontal	Source	*df*	*F*	*p*	ηp^2^
	Menstrual cycle phase	1	3.194	0.088	0.127
	Mid-luteal baseline	1	3.418	0.078	0.134
	Early follicular baseline	1	2.730	0.113	0.110
	Error (cycle)	27			
Temporal	Source	*df*	*F*	*p*	ηp^2^
	Menstrual cycle phase	1	4.712	0.04	0.159
	Mid-luteal baseline	1	0.100	0.754	0.004
	Early follicular baseline	1	0.877	0.358	0.034
	Error (cycle)	29			
Parietal	Source	*df*	*F*	*p*	ηp^2^
	Menstrual cycle phase	1	4.859	0.042	0.222
	Mid-luteal baseline	1	0.128	0.725	0.007
	Early follicular baseline	1	1.718	0.207	0.092
	Error (cycle)	29			

**Table 4:** The results of repeated-measures ANCOVAs with menstrual cycle phase (early follicular vs. mid-luteal) as the within-subjects source of variance, baseline EEG activity (without cognitive load) during both phases of the menstrual cycle as covariates, and laterality indices as the dependent variables during the verbal fluency task.

Task: Verbal Fluency					
Frontal	Source	*df*	*F*	*p*	ηp^2^
	Menstrual cycle phase	1	5.753	0.023	0.166
	Mid-luteal baseline	1	1.614	0.214	0.053
	Early follicular baseline	1	0.964	0.334	0.032
	Error (cycle)	29			
Temporal	Source	*df*	*F*	*p*	ηp^2^
	Menstrual cycle phase	1	4.275	0.049	0.141
	Mid-luteal baseline	1	0.809	0.377	0.030
	Early follicular baseline	1	0.390	0.538	0.015
	Error (cycle)	29			
Parietal	Source	*df*	*F*	*p*	ηp^2^
	Menstrual cycle phase	1	10.467	0.003	0.279
	Mid-luteal baseline	1	0.288	0.596	0.011
	Early follicular baseline	1	1.894	0.180	0.066
	Error (cycle)	29			

## Data Availability

The data presented in this study are available from the corresponding author on request. The datasets generated and analyzed in the present study are not publicly available due to their size and complexity. In particular, EEG research yields a substantial volume of raw data distributed across a large number of individual files, while the behavioral data are organized and coded in a different format. These characteristics render the direct deposition of the dataset in a public repository impractical. Nevertheless, the data that support the findings of this study are available from the corresponding author upon reasonable request. To ensure clarity and relevance, requests should specify the type and scope of the data required.

## Supplementary Materials

The following supporting information can be downloaded at https://www.mdpi.com/article/10.3390/brainsci15111141/s1, Table S1. The results of repeated measures ANCOVAs, with menstrual cycle phase as a within-subject source of variance, order of testing (early follicular vs. mid-luteal first) as a between-subject source of variance and performance on verbal tasks as dependent variables; Table S2. Adjusted *p*-values after Benjami-Hochberg correction for hypotheses expecting significance.
