# Supplementary material for: Menstrual Cycle Modulation of Verbal Performance and Hemispheric Asymmetry
Source: Brain Sci. 2025 Oct 24;15(11):1141. doi: 10.3390/brainsci15111141 (PMC12650095; doi:10.3390/brainsci15111141)
Supplement: Supplementary file 1 [file brainsci-15-01141-s001.zip › brainsci-3872568-supplementary.pdf]

Supplementary material, Hromatko & Tadinac: Menstrual cycle modulation of verbal performance and hemispheric asymmetry

Table S1. The results of repeated measures ANCOVAs, with menstrual cycle phase as a within-subject source of variance, order of testing (early follicular vs. mid-luteal first) as a between-subject source of variance performance on verbal tasks as dependent variables

	Source	*df*	*F*	*p*	ηp^2
**Verbal fluency**	**Menstrual cycle phase**	**1**	**4.766**	**0.037**	**0.141**
	Order of testing	1	0.057	0.813	0.002
	Fatigue * mid-luteal	1	0.135	0.688	0.006
	Fatigue * early follicular	1	2.487	0.126	0.079
	Error(cycle)	29			
	Source	*df*	*F*	*p*	ηp^2
**Semantic decision**	Menstrual cycle phase	**1**	**5.499**	**0.026**	**0.164**
	Order of testing	1	0.007	0.934	0.001
	Fatigue * mid-luteal	1	0.748	0.395	0.026
	Fatigue * early follicular	1	0.024	0.879	0.001
	Error(cycle)	29			
	Source	*df*	*F*	*p*	ηp^2
**Verbal reasoning**	Menstrual cycle phase	**1**	**0.878**	**0.357**	**0.029**
	Order of testing	1	0.104	0.750	0.004
	Fatigue * mid-luteal	1	0.025	0.875	0.001
	Fatigue * early follicular	1	1.16	0.29	0.038
	Error(cycle)	29			

Table S2. Adjusted p-values after Benjami-Hochberg correction for hypotheses expecting significance

**Task, location**	**Original *p***	**Adjusted *p***
Verbal fluency, frontal	0.023	0.069
Verbal fluency, temporal	0.049	0.0588
Verbal fluency, parietal	0.003	0.018
Semantic decision, frontal	0.088	0.088
Semantic decision, temporal	0.04	0.08
Semantic decision, parietal	0.042	0.063
